# A cross-sectional study of the use of graduated compression stockings at two top end hospitals in Darwin, Australia

**DOI:** 10.1016/j.jvsv.2026.102528

**Published:** 2026-05-18

**Authors:** Jennifer M. Byrnes, Robert J. Commons, Mark J. Hamilton, Jonathon Bowden, That Ton, David Toro Tole, Sami Quader, Yuzhi Wang, Sherin Vincent, Lucy Jenkins, Madelyne Lu, Jillian Wong, Rachel Yun, Joyce Sun, Aaron Gordon, Gabrielle B. McCallum

**Affiliations:** a Child and Maternal Health Division, Menzies School of Health Research, Charles Darwin University, Tiwi, Northern Territory, Australia; b Division of Surgery and Critical Care, Royal Darwin and Palmerston Hospital, Tiwi, Northern Territory, Australia; c Global and Tropical Health Division, Menzies School of Health Research and Charles Darwin University, Tiwi, Northern Territory, Australia; d General and Subspecialty Medicine, Grampians Health, Ballarat, Victoria, Australia; e Department of Vascular and Endovascular Surgery, Royal Darwin and Palmerston Hospital, Darwin, Northern Territory, Australia; f Flinders University School of Medicine, Flinders University Medicine Program, Bedford Park, Australia; g School of Nursing and Midwifery, Charles Darwin University, Casuarina, Northern Territory, Australia

**Keywords:** Graduated compression stockings, Deep vein thrombosis, Venous thromboprophylaxis, Pulmonary embolism, Venous thromboembolism

## Abstract

**Background:**

Venous thromboembolism (VTE) is the main cause of hospital-acquired adverse events globally, affecting approximately 10 million people annually. Methods of VTE prophylaxis include anticoagulants and/or mechanical prophylaxis, such as graduated compression stockings (GCS).

**Objective:**

This study aimed to determine whether (a) GCS are used appropriately for VTE prophylaxis and (b) GCS are fitted correctly or cause adverse events.

**Methods:**

A cross-sectional study of adults aged >18 years admitted to acute wards of two Darwin public hospitals was undertaken in May 2024. Medical records were examined to identify if GCS were prescribed and if any contraindications to GCS were present. Participants who were wearing or had worn GCS within the last 24 hours were assessed for GCS usage, prescription of GCS, the presence of contraindications to GCS, correct fitting of GCS, and the presence of adverse events.

**Results:**

Of 398 admitted participants, GCS were prescribed in 49 (13%) and GCS were being used in 77 (19%). A total of 46 (12%) participants consented to assessment of GCS fit. Thirteen participants had contraindications for GCS use, 6 (13%) potential and 7 (15%) absolute, with 4 (31%) having adverse events associated with GCS use. Measurements of the knee, calf, and ankle found that only 6 (14%) of 43 participants were wearing GCS that fitted correctly according to the manufacturer's recommendations, and leg measurements were not taken in 20 (44%) participants before GCS application. There were a wide variety of foot sizes in participants wearing each of the different sized GCS, with reduced correlation between foot width and knee (ρ = 0.26) or calf circumference (ρ = 0.27).

**Conclusions:**

The current study found reduced compliance with measures designed to enable correct GCS fitting at each stage of the fitting cascade. Foot sizes varied with other leg measurements, suggesting a greater need for personalized fitting approaches. There is a need for a systematic approach to education and fitting of GCS in health care services to ensure their correct and safe fitting.

**Article Highlights**

• **Type of Research:** Cross-sectional study
• **Key Findings:** Among 398 participants, graduated compression stockings (GCS) were prescribed in 49 (13%) and used in 77 (19%). Forty-six (12%) consented to having GCS fit assessed. Thirteen (28%) had contraindications to GCS use, and four (31%) experienced related adverse events. Only six of the 43 participants (14%) were wearing GCS that fitted correctly.
• **Take Home Message:** Reduced compliance with measures designed to enable correct GCS fitting at each stage of the fitting cascade was observed, potentially decreasing GCS efficacy and safety among patients. There is a need for a systematic approach to use, education, and fitting of GCS in tertiary settings to ensure patient safety.

Venous thromboembolism (VTE) is the main cause of hospital-acquired adverse events globally,^1^ affecting approximately 10 million people each year.^2^ The annual incidence of VTE ranges from 115 to 269 per 100,000,^2^, ^3^, ^4^ with an estimated financial burden of AU$1.72 billion in Australia,^5^ £640 million in the United Kingdom,^6^ and US$15.5 billion in the United States per annum.^7^

Without thromboprophylaxis, the risk of VTE for surgical patients in hospital is approximately 30% (29% for deep vein thromboses [DVT] and 3% for pulmonary emboli [PEs]), compared with 24% for DVTs and 1% for PEs in medical patients.^8^^,^^9^ There is an associated 30-day mortality rate of 3% for DVTs and 31% for PEs.^10^ Approximately 50% of all VTE events occur during hospitalization for an acute medical illness or surgery.^11^ The risk of VTE in patients undergoing surgery increases with age, with patients older than 70 years having a 65% risk of a VTE event after surgery without prophylaxis.

In Australia, between 2010 and 2013, a population-based study found that the incidence rate of hospital-acquired VTE was 9.7 per 1000 admissions that lead to death in 4.3% of people.^12^ Furthermore, 10% of all Australian hospital deaths can be attributed to VTE annually.^13^ To reduce the burden and mortality of hospital-acquired VTE, the Australian National Safety and Quality commission developed a clinical care standard in 2020 focusing on identifying VTE and prevention strategies including thromboprophylaxis,^13^ and suggested that appropriate implementation of thromboprophylaxis can reduce VTE incidence by as much as 70% in surgical and medical patients.^13^^,^^14^

Recommended methods of VTE prophylaxis include monotherapy or combination therapy using anticoagulants and/or mechanical prophylaxis, such as graduated compression stockings (GCS). GCS have been a cornerstone of prophylaxis for many years; however, increasing questions about their relative benefit have been raised.^15^ GCS sizes do not fit all leg or foot types, and fitting does not incorporate foot measurements, meaning a person with a slender leg and large foot may end up wearing GCS that are too small for the foot or vice versa. Incorrect fitting can result in adverse events, including pressure injuries or falls, or alteration of the lower limb pressure gradient reducing the intended thromboprophylactic effect.^16^, ^17^, ^18^, ^19^

Foot deformities may further increase the risk of uneven pressure at the foot section of the GCS. Most studies assessing the efficacy of GCS have been undertaken in predominantly Caucasian populations in high-income countries.^8^ Yet, to the best of our knowledge, the extent of different leg profiles and how they affect the efficacy of GCS has not been assessed by any study.^8^ Importantly, a contraindication stated by GCS manufacturers is to avoid use in the presence of an unusual shaped leg or extreme deformity that would prevent correct fit.^20^ This directive becomes ambiguous when the natural leg shape of a person is normal for one population but differs to other populations. For example, older participants may be at an increased risk of having dependent lower limb edema and foot deformities such as claw toe and hallux valgus (bunions). This issue was highlighted in a study of 19 older participants using GCS, with all reporting discomfort on the hallux valgus, women reporting the foot section to be too large, and a mix of participants finding the GCS too tight in the arch region.^21^

Overall, the most efficacious option to prevent a VTE event and the relative risks and benefits of different thromboprophylaxis techniques remains uncertain.^8^^,^^22^, ^23^, ^24^ To better inform GCS implementation and safety for VTE prophylaxis, this cross-sectional study aimed to assess the cascade of GCS care including prescribing, appropriate use, safety, and fitting of GCS with participants admitted to two hospitals in the Top End of the Northern Territory (NT), Australia, to determine whether (a) GCS are used appropriately for VTE prophylaxis and (b) GCS are fitted correctly or cause adverse events.

## Methods

### Setting

This cross-sectional study was undertaken among adult (>18 years) patients in the acute wards of two Darwin public hospitals over 7 days during May 2024. Royal Darwin Hospital is a 360-bed tertiary teaching hospital providing emergency care, surgical, medical, obstetric, and pediatric services plus specialist outpatient services. Palmerston Regional Hospital, a 116-bed hospital, provides medical services, low acuity surgical procedures, and emergency services. These hospitals provide services to an estimated population of 167,700 people from the Top End of the NT.^25^

### Study design and participants

This study consisted of two parts (part A and part B). Eligibility for part A included adult participants admitted to one of the two hospitals. Participants were excluded if their medical records were unavailable. Eligibility for part B included participants who were wearing or had worn GCS within the last 24 hours. Participants were excluded if they were unavailable for assessment or were isolated due to COVID-19.

The research team were trained, and the study protocol and data collection forms were validated before the study commenced to ensure reliability and replication of results on assessment. A standardized data collection tool was used via REDCap (Research Electronic Data Capture).

The Human Research Ethics Committee of NT Health and Menzies School of Health Research (HREC 2023-4549) and Charles Darwin University (H23047) approved this study. A waiver for consent was approved for part A, and written informed consent was obtained for participants eligible for part B.

### Data collection

Medical charts of all participants were reviewed for demographics, medical history, contraindications for GCS, adverse events, and details of GCS prescription. Contraindications for GCS use were collected from the medical admission notes. GCS prescription data including presence, location, prescriber, and reason for GCS were collected from medical records and/or the electronic medication chart.

Absolute contraindications were defined as the presence of lower limb ischemia, peripheral arterial disease, presence of gangrene, tissue loss of lower limb or toes, peripheral neuropathy, or a lower limb pressure injury. Lower limb ischemia or peripheral arterial disease was defined as the (1) absence of pulses on clinical examination, (2) documented symptoms of lower limb rest pain or claudication, (3) radiologically significant arterial disease confirmed by imaging, (4) ankle-brachial index <0.7 or toe pressures <50 mm Hg, or (5) previous arterial intervention. Potential contraindications for GCS use included prior lower limb amputation, diagnosis of Charcot arthropathy, and the presence of other foot deformities, for example, hammer toes, bunions, congenital deformities, or traumatic injury changes.

For part B, data were collected across five domains: (1) type of thromboprophylaxis implemented, (2) GCS fitting, (3) contraindications, (4) adverse events, and (5) participants' perceptions of GCS use, via a short survey. Assessors underwent training based on a standardized protocol before the study and undertook assessments across both sites.

After written consent, GCS fit and sizing were assessed by physical examination by two researchers. Visual inspection involved the initial review of the GCS in place from the bedside before conducting a physical examination. At this point, the researchers gave an initial determination if GCS appeared to be fitted appropriately. Physical examination involved closer inspection by lifting and moving the leg to gain a 360° view of the leg and foot. This enabled the researcher to assess if the heel, foot, and knee sections were located at appropriate leg landmarks, if the stocking was rolled or folded over the foot or leg, if GCS were loose in the leg section with a tight foot, or a tight leg section with looseness around the foot, if the foot section was too long or too short, or if excess stocking was present at the popliteal crease, ankle, or toes.

Three anatomical locations (below knee, calf, and ankle circumferences) were used to size GCS. After visual inspection by the research team, the GCS were removed, and the lower limb measured at these points to determine if the size of the stocking in situ was correct. Risk factors and potential contraindications to GCS were then assessed, including the presence of posterior tibial and dorsalis pedis pulses, leg deformities, and foot deformities.

Adverse events caused by GCS were assessed by physical examination of the legs and feet and then correlated with participant history and medical record review. The association of the adverse event with GCS usage was categorized as unlikely, possible, probable, or definite, and the severity of the adverse event was defined as mild, moderate, severe, or life-threatening according to the Australian Commission on Safety and Quality in Health Care.^26^

Data were entered directly into standardized data collection forms on a password-protected REDCap database by members of the research team.

### Outcomes

Outcomes assessed were GCS usage, prescription of GCS, the presence of contraindications to GCS, correct fitting of GCS, leg measurement before GCS application, the presence of adverse events, and frequency of combination of thromboprophylaxis therapy present.

### Statistical analysis

Data were analyzed using STATA (version 17; Stata Corp Station). Summary statistics are presented as either median (interquartile range [IQR]: 25th-75th percentile) or mean (standard deviation) for continuous data, depending on distribution, and as frequency (percentage) for categorical data.

## Results

### Part A

A total of 398 participants were included. The median age was 62 years (IQR: 43-75 years), 229 (58%) were male, 242 (61%) identified as non–First Nations, and 226 (57%) were admitted to the medical ward (Table I). GCS were prescribed in 49 (13%) participants, and 33 (67%) prescriptions were issued by doctors, with surgery stated as the reason for prescription in 23 (47%) participants. Of these 49 participants, 3 (6%) had absolute contraindications, and 1 (2%) had potential contraindications for GCS use (Supplementary Table I, online only).

**Table I:** Participant demographics for parts A and B

Demographics	All participants (part A) (n = 398)	Participants wearing GCS (part B) (n = 46)
Age, years	62 (43-75)	51.5 (38-66)
Sex		
Female	169 (42)	17 (37)
Male	229 (58)	29 (63)
Ethnicity		
First Nations	156 (39)	10 (22)
Non–First Nations	242 (61)	36 (78)
Division		
Surgical	153 (38)	33 (72)
Medical	226 (57)	9 (19)
Obstetric	19 (5)	4 (9)
Residency		
Urban	279 (70)	28 (61)
Rural	111 (28)	14 (30)
Interstate	8 (2)	4 (9)

*GCS*, Graduated compression stockings.

Data presented as median (interquartile range) or number (%).

### Part B

Of the 398 participants in part A, only 77 (19%) were eligible for part B. These 77 participants were more likely to be younger, identify as non–First Nations, reside in urban areas, and were admitted under the surgical division compared with participants who were not wearing GCS (Supplementary Table I, online only).

After excluding 31 of 77 (8%) participants who did not consent or were isolated due to COVID-19, 46 of 398 (12%) participants were included in part B. Their median age was 51.5 years (IQR: 38-66 years), 17 (63%) were male, and 36 (78%) identified as non–First Nations (Table I). Of the 46 participants, GCS were prescribed in 15 (33%), with surgery in 7 (47%) being the primary reason for prescription (Table II). Most participants (37 of 46, 80%) had combination thromboprophylaxis with GCS and anticoagulants. Five (11%) had GCS as monotherapy, two (4%) had dual GCS and intermittent calf pumps, and two (4%) had triple therapy of GCS, intermittent calf pumps, and anticoagulants. No foot pumps were in use, although these were available at both hospitals.

**Table II:** Prescription of graduated compression stockings (*GCS*)

GCS order	All participants (n = 398)	Participants wearing GCS^a^ (n = 46)
GCS prescribed/ordered, No. (%)	49/398 (13)	15/46 (33)
Location of order, No. (%)		
Electronic medicine chart	20/49 (41)	3/15 (20)
Medical records (paper and electronic)	20/49 (41)	6/15 (40)
Other	9/49 (18)	6/15 (40)
Person who ordered, No. (%)		
Doctor	33/49 (67)	7/15 (47)
Nurse	13/49 (27)	6/15 (40)
Other	3/49 (6)	2/15 (14)
Reason for order, No. (%)		
Surgery	23/49 (47)	7/15 (47)
Medical condition	4/49 (8)	1/15 (7)
Pregnancy	1/49 (2)	0/15 (0)
Immobility	2/49 (4)	1/15 (7)
Not stated	18/49 (37)	5/15 (33)
Other	1/49 (2)	1/15 (7)

a Participants wearing GCS who consented for physical assessment.

Potential contraindications for GCS use were present in six (13%) participants: five (11%) with bunions and one (2%) with a great toe amputation. A further seven (15%) participants had absolute contraindications for GCS use, including five (11%) with absent foot or leg pulses, one (2%) with peripheral neuropathy, and one (2%) with a lower limb pressure injury (Supplementary Table II, online only). Of 13 participants with contraindications to GCS use, 3 (23%) had a GCS prescription.

Nine adverse events were identified from seven participants as probably or definitely related to GCS use. Six (13%) reported leg pain or burning of the heels (one had resolved before study assessment because of the GCS size being changed due to leg pain), two (4%) had skin tears, and one (2%) had a lower limb pressure injury. Four of the 13 (31%) participants with potential or absolute contraindications to GCS use had adverse events. Of the seven participants who experienced adverse events, five (71%) were from the surgical division, all identified as non–First Nation, four (57%) were female, and four (57%) did not have a GCS prescription.

On inspection by the research team before measurement, 18 of 46 (39%) participants had concerns with fitting of GCS (Supplementary Table III, online only).

**Fig 1:**
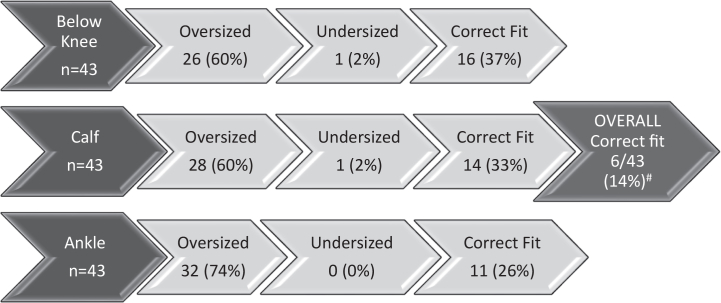
Assessment of fit of graduated compression stockings (GCS) by measurement at three anatomical locations. NB: three participants with unknown measurements were excluded from assessment; #fit was based on circumference at the site measured as per manufacturers recommendations; #overall fit at all three locations simultaneously was only assessed as correct if measurements were within the correct range for all anatomical locations of the leg. Given that in some participants, there may have been some locations that were oversized and others that were undersized, overall correct fit is lower than the sum of the individual location correct fit number.

Twenty (44%) participants were not measured by hospital staff before original GCS application. Lower limb measurements were available for all 46 participants; however, three were excluded from the analysis of correct fit as current GCS size could not be identified. Comparison of measurements of the knee, calf, and ankle with the GCS size being worn found that only 6 of 43 (14%) participants were wearing GCS that fitted correctly according to the manufacturer's recommendations (Fig 1). Two (33%) were female, and all were non–First Nations. Of the six participants who had GCS fitted correctly, one (17%) had a potential contraindication, four (67%) had absolute contraindications, and two (33%) experienced adverse events due to GCS. Furthermore, most participants wearing GCS were oversized at each anatomical location (Fig 1).

Table III demonstrates the correct fitting percentage by manufacturer size at each anatomical site. Substantial variability in the range of measurements was observed for each site and stocking size. Foot sizing varied considerably from 7.5 to 13.4 cm in width and 20.5 to 29 cm in length. There were a wide variety of foot sizes in participants wearing each of the different sized GCS. Although foot length was correlated moderately with knee (ρ = 0.52), calf (ρ = 0.47), and ankle (ρ = 0.70) circumference, foot width was not correlated significantly with knee (ρ = 0.26) or calf circumference (ρ = 0.27). Some individuals with smaller recorded circumferences had longer and wider feet (Supplementary Fig, online only).

**Table III:** Comparison of actual to recommended fit of graduated compression stockings (*GCS*) by size in 43 participants wearing GCS

GCS size worn	Median (range) in participants, cm	Manufacturer recommendation, cm	Correct fit, No. (%)
Below knee			
Small	30.7 (23-37.5)	29-37	5/8 (63)
Medium	34 (29.5-38.5)	33-43	8/14 (57)
Large	35.3 (31.2-46)	37-47	3/17 (18)
X large	36.3 (31-41)	43-54	0/4 (0)
Calf			
Small	29.5 (21.5-38.4)	30-38	3/8 (38)
Medium	34.3 (26.8-39)	34-44	8/14 (57)
Large	35.4 (27-48)	38-48	3/17 (18)
Extra-large	33 (30-38.5)	44-55	0/4 (0)
Ankle			
Small	19.6 (15-22.3)	20-22	3/8 (38)
Medium	22.6 (20.6-26)	23-25	5/14 (36)
Large	23.1 (20-27.5)	26-28	3/17 (18)
Extra-large	23.4 (21-26)	29-31	0/4 (0)

Three participants were excluded from assessment of correct fit as the GCS size they were wearing was unable to be determined. Correct fit at all three sites was found in 1 of 8 (13%) participants wearing small, 3 of 14 (21%) participants wearing medium, 2 of 17 (12%) participants wearing large, and 0 of 4 (0%) participants wearing extra-large.

### Participant perceptions

Thirty-five participants (76%) stated that they knew why they were asked to wear GCS. Most (n = 38, 83%) found the stockings comfortable, with 32 (70%) liking wearing them but 7 (15%) preferring to use a machine or medicine instead of wearing GCS. One participant described GCS as causing shame or embarrassment.

## Discussion

**Fig 2:**
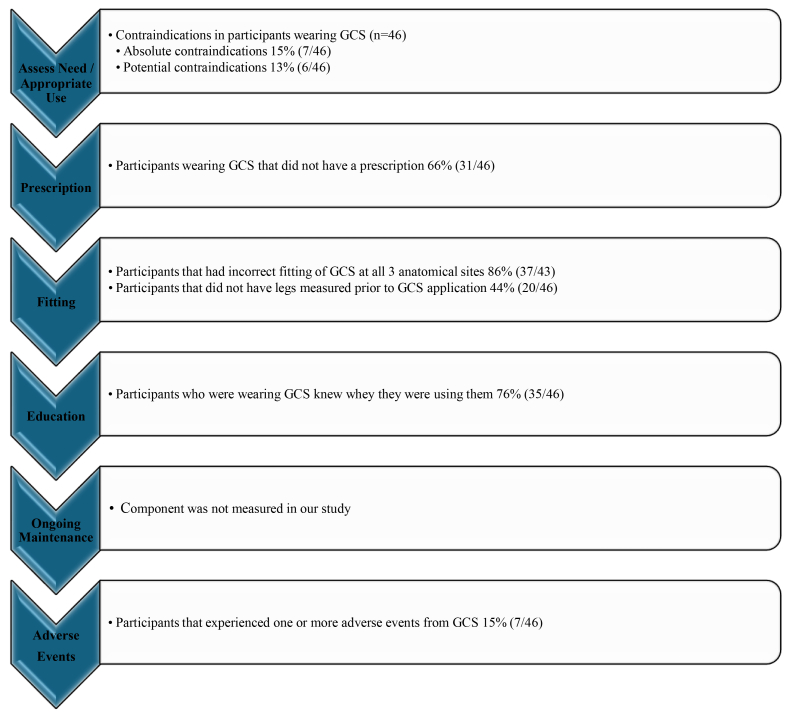
Compliance of implementing graduated compression stockings (*GCS*) as per fitting cascade.

VTE prophylaxis is critical to prevent life-threatening complications of hospital admission. GCS are an important tool for VTE prophylaxis; however, increasing questions about their relative benefit are being raised,^15^ and standardized sizing means some individuals may be fitted incorrectly leading to reduced efficacy or potential harm. This multisite cross-sectional study assessed the GCS fitting cascade, including prescription, appropriate use, fitting, and safety of GCS use in hospital inpatients. It found a relatively low prevalence of use across inpatients and identified reduced levels of compliance at each level of the overall GCS fitting cascade (Fig 2). This included lack of GCS prescription in 67% of participants wearing GCS, contraindications in 28% wearing GCS, no documented leg measurements before GCS application in 44%, and incorrect fitting according to the manufacturer's recommendations in 86%.

Measurement of legs before fitting of GCS is a cornerstone to ensure that they provide effective VTE prophylaxis. GCS or antiembolic stockings are specifically designed for patients resting in bed for VTE prophylaxis and produce compression from 15 to 18 mm Hg; however, they become ineffective with mobilization due to design for use in supine patients.^20^^,^^23^ Only 56% of GCS patients were measured in this study. Furthermore, of those wearing GCS, only 14% were correctly sized using measurements at their knee, calf, and ankle according to the manufacturer's recommendations, highlighting difficulty with correct sizing, even with prior measurement. Our data differ from previous studies that demonstrated that correct measurement of legs avoided complications and adverse events,^20^^,^^27^ and that accurate measurements as per the manufacturer's instructions should enable appropriate fit.^20^^,^^27^ For example, in 1984, Turner et al^28^ indicated that nine different sizes of GCS could fit 95% of the population. Since 2013, however, there has been increased debate about the appropriate fit and thromboprophylactic efficacy of GCS^16^ with a study of critically ill patients finding pressure injuries in 31% wearing GCS.^29^

In our study, even if GCS were sized correctly on visual inspection, closer physical examination revealed fitting issues, such as bunching behind the knee or around the foot, or excess stocking around the ankle and toes, increasing the risk of adverse events, such as pressure injuries or falls (eg, nine adverse events occurring in this study). Furthermore, correct fitting of GCS is essential to achieve the mechanical compression needed to prevent DVTs. This ensures a sufficient circumferential and longitudinal pressure gradient to augment venous flow to the deep venous system while the patient is immobile.^16^^,^^30^

Even in optimally fitted GCS, individual anatomical variation may lead to reduced efficacy and adverse events. This was highlighted in a 2018 review that found that no study had addressed how variable leg profiles may limit the efficacy of GCS.^8^ To the best of our knowledge, the relative size of the foot profile is not formally measured during GCS fitting and has not been considered by GCS trials to date, nor is it a formal component of GCS fitting. In people with a relatively large foot, if GCS are fitted correctly to the calf and ankle, the foot may be more prone to pressure injuries over bony prominences.^19^^,^^29^ Our study found that although knee, calf, and ankle circumferences were correlated to foot length, correlation to foot width was reduced (Supplemental Fig, online only). This may place people using otherwise correctly fitted GCS at increased risk of pressure injuries on their feet if GCS are overly tight or increased risk of slipping if GCS are loose around their feet. In contrast, if the ankle is relatively small compared with the calf, GCS may not develop the pressure gradient required to create effective VTE prophylaxis. As most of our study participants had incorrectly fitted GCS, with >66% being oversized, it is likely that they would have reduced or no VTE prophylactic effect.

There is currently no standardized location for GCS prescription in the hospitals we undertook the study, and prescription of GCS was only present in 33% of participants assessed. GCS were regularly used without a prescription or in the presence of a contraindication, suggesting a lack of systematic fitting, increasing the risk of adverse events.

**Fig 3:**
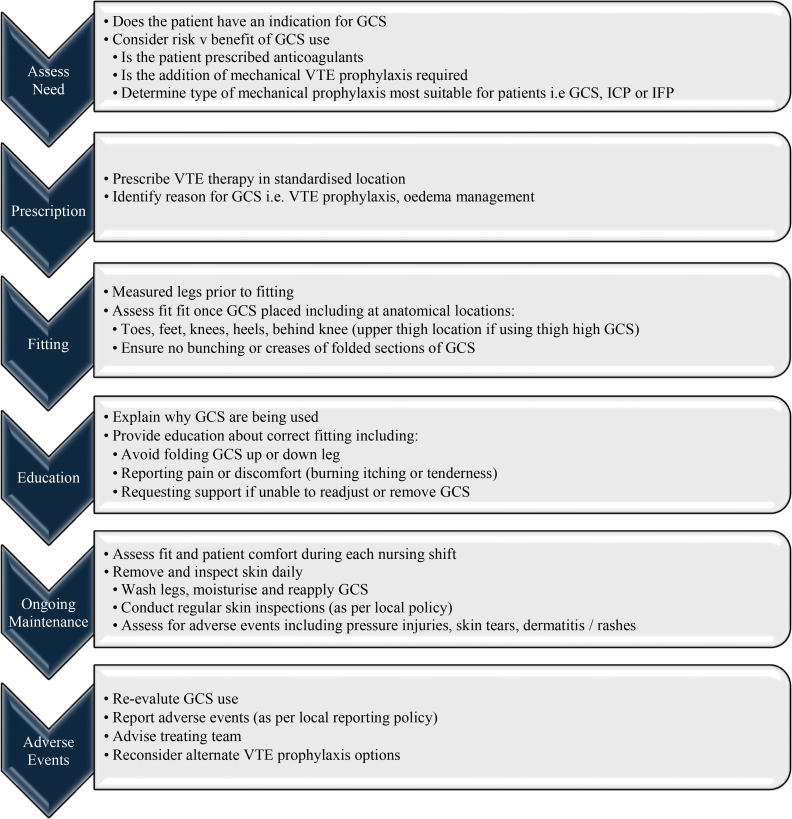
Graduated compression stocking fitting cascade flow diagram. *VTE*, Venous thromboembolism.

Contraindications for GCS use vary from potential to absolute, with a variable degree of risk for subsequent adverse events. In this study, six participants (13%) wearing GCS had potential contraindications and a further seven had absolute contraindications for GCS. In addition, seven participants wearing GCS experienced nine adverse events including 31% of the participants with identified contraindications to GCS. To mitigate risk, it is essential that staff identify risk factors for GCS use before application through history and examination. Training of staff in safe GCS use is essential to ensure appropriate prescription, use, application, and sizing of GCS.^31^ A standardized GCS fitting cascade would provide a structure for this process and enable regular monitoring and evaluation (Fig 3). Unfortunately, GCS has a reputation of being harmless as they are not perceived as a medical intervention;^32^ however, complications of GCS incorrect use or application can be serious.

This study had several limitations. The number of participants using GCS was low (n = 77, 19%), preventing a detailed analysis of fit, sizing, adverse events, and contraindications that adjusted for confounders. This may be due to ongoing academic debate about the efficacy of GCS leading to reduced clinical uptake, together with increased awareness of the potential for adverse effects. For example, within the hospitals of this study, an informal education campaign within the surgery disciplines has advocated for the avoidance of GCS in the presence of neuropathy or lower limb ischemia. Furthermore, the anecdotal development of heel pressure injuries in patients wearing GCS in the intensive care unit has led to a reduction in their use, which was further supported by similar published reports of pressure injuries in the intensive care unit setting.^32^

## Conclusions

The current study found reduced compliance with measures designed to ensure correct use of GCS at each level of the GCS fitting cascade, including a small percentage of patients with correctly fitted GCS. In addition, foot width was not well correlated with other leg measurements, highlighting the potential for an increase in adverse events despite adequate fitting. The results highlight the need for further robust studies to show evidence for or against GCS use in diverse populations with varied foot and leg profiles. They also highlight the need for a standardized systematic approach to education and fitting of GCS that considers each step in their use from the decision to implement GCS to their ongoing monitoring.

## Author Contributions

Conception and design: JMB, RC, MH, GM

Analysis and interpretation: JMB, RC, MH, GM

Data collection: JMB, RC, JB, TT, DT, SQ, YW, SV, LJ, ML, JW, RY, JS, AG

Writing the article: JMB, RC, MH, GM

Critical revision of the article: JMB, RC, MH, JB, TT, DT, SQ, YW, SV, LJ, ML, JW, RY, JS, AG, GM

Final approval of the article: JMB, RC, MH, JB, TT, DT, SQ, YW, SV, LJ, ML, JW, RY, JS, AG, GM

Statistical analysis: Not applicable

Obtained funding: Not applicable

Overall responsibility: GM, RC

## Funding

R.J.C. is supported by an Australian 10.13039/501100000925National Health and Medical Research Council Emerging Leader Investigator Grant (1194702).

## Disclosures

None.

## References

[1] Rabinovich A., Ducruet T., Kahn S.R. (2018). Development of a clinical prediction model for the postthrombotic syndrome in a prospective cohort of patients with proximal deep vein thrombosis. J Thromb Haemost.

[2] Raskob G.E., Angchaisuksiri P., Blanco A.N. (2014). Thrombosis: a major contributor to global disease burden. Arterioscler Thromb Vasc Biol.

[3] Wenger N., Sebastian T., Engelberger R.P., Kucher N., Spirk D. (2021). Pulmonary embolism and deep vein thrombosis: similar but different. Thromb Res.

[4] Tran H., Gibbs H., Merriman E. (2019). New guidelines from the thrombosis and Haemostasis Society of Australia and New Zealand for the diagnosis and management of venous thromboembolism. Med J Aust.

[5] Rickard C.M., Marsh N., Webster J. (2015). Securing all intravenous devices effectively in hospitalised patients--the SAVE trial: study protocol for a multicentre randomised controlled trial. BMJ open.

[6] Cohen A., Pieter D., Marchant N. (2011). The efficacy and safety of pharmacological prophylaxis of VTE following elective knee or hip replacement: systematic review and network meta-analysis. Blood.

[7] Cundiff D.K. (2004). Anticoagulation therapy for venous thromboembolism. MedGenMed.

[8] Sachdeva A., Dalton M., Lees T. (2018). Graduated compression stockings for prevention of deep vein thrombosis. Cochrane database Syst Rev.

[9] Eppsteiner R.W., Shin J.J., Johnson J., van Dam R.M. (2010). Mechanical compression versus subcutaneous heparin therapy in postoperative and posttrauma patients: a systematic review and meta-analysis. World J Surg.

[10] Søgaard K.K., Schmidt M., Pedersen L., Horváth-Puhó E., Sørensen H.T. (2014). 30-year mortality after venous thromboembolism: a population-based cohort study. Circulation.

[11] Schünemann H.J., Cushman M., Burnett A.E. (2018). American Society of Hematology 2018 guidelines for management of venous thromboembolism: prophylaxis for hospitalized and nonhospitalized medical patients. Blood Adv.

[12] Stubbs J.M., Assareh H., Curnow J., Hitos K., Achat H.M. (2018). Incidence of in-hospital and post-discharge diagnosed hospital-associated venous thromboembolism using linked administrative data. Intern Med J.

[13] Australian Commission on Safety and Quality in Health Care (2020). Quality NSa.

[14] Hibbert P.D., Hannaford N.A., Hooper T.D. (2016). Assessing the appropriateness of prevention and management of venous thromboembolism in Australia: a cross-sectional study. BMJ Open.

[15] Turner B.R.H., Machin M., Salih M. (2024). An updated systematic review and meta-analysis of the impact of graduated compression stockings in addition to pharmacological thromboprophylaxis for prevention of venous thromboembolism in surgical inpatients. Ann Surg.

[16] Macintyre L., Kent K., McPhee D. (2013). Do anti-embolism stockings fit our legs? Leg survey and data analysis. Int J Nurs Stud.

[17] Thompson A., Walter S., Brunton L.R. (2011). Anti-embolism stockings and proximal indentation. Br J Nurs.

[18] Patel N., Khakha R., Gibbs J. (2013). Review article: anti-embolism stockings. J Orthopaedic Surg.

[19] Rabe E., Partsch H., Morrison N. (2020). Risks and contraindications of medical compression treatment – a critical reappraisal. An international consensus statement. Phlebology.

[20] Lim C.S., Davies A.H. (2014). Graduated compression stockings. CMAJ.

[21] Goetz J., Kaisermayer E., Haase H., Jünger M., Riebe H. (2019). Better wearing comfort of knee-length elastic compression stockings with an interface pressure of 18-21 mmHg compared to 23-32 mmHg in elderly people after a one day trial – influence on foot deformities, rheumatism and arthritis. Clin Hemorheol Microcirc.

[22] Khatri A., Machin M., Vijay A., Salim S., Shalhoub J., Davies A.H. (2021). A review of Current and future antithrombotic strategies in surgical patients—leaving the graduated compression stockings behind?. J Clin Med.

[23] Machin M., Peerbux S., Whittley S. (2023). Examining the benefit of graduated compression stockings in the prevention of hospital-associated venous thromboembolism in low-risk surgical patients: a multicentre cluster randomised controlled trial (PETS trial). BMJ open.

[24] Suna K., Herrmann E., Kröger K. (2020). Graduated compression stockings in the prevention of postoperative pulmonary embolism. A propensity-matched retrospective case-control study of 24 273 patients. Ann Med Surg (London).

[25] Territory D.O.H.N., Darwin N.T. (2023). Department of Health NT. NT Health Annual Report 2022-23.

[26] Health D.O., Australian Commission on Safety and Quality in Health Care (2021). Elizabeth street.

[27] Winslow E.H., Brosz D.L. (2008). Graduated compression stockings in hospitalized postoperative patients: correctness of usage and size. Am J Nurs.

[28] Turner G.M., Cole S.E., Brooks J.H. (1984). The efficacy of graduated compression stockings in the prevention of deep vein thrombosis after major gynaecological surgery. Br J Obstet Gynaecol.

[29] Basford J.R. (2002). The law of laplace and its relevance to contemporary medicine and rehabilitation. Arch Phys Med Rehabil.

[30] Benkö T., Cooke E.A., McNally M.A., Mollan R.A. (2001). Graduated compression stockings: knee length or thigh length. Clin orthopaedics Relat Res.

[31] Xu Y., Wang W., Zhao J., Wang J., Zhao T. (2019). Knowledge, attitude, and practice of healthcare professionals toward clinically applying graduated compression stockings: results of a Chinese web-based survey. J Thromb Thrombolysis.

[32] Hobson D.B., Chang T.Y., Aboagye J.K. (2017). Prevalence of graduated compression stocking–associated pressure injuries in surgical intensive care units. J Crit Care.

