# Supplementary material for: A cross-sectional study of the use of graduated compression stockings at two top end hospitals in Darwin, Australia
Source: J Vasc Surg Venous Lymphat Disord. 2026 May 18;14(5):102528. doi: 10.1016/j.jvsv.2026.102528 (PMC13285706; doi:10.1016/j.jvsv.2026.102528)
Supplement: Supplementary Tables and Figures [file mmc1.docx]

**Supplementary Tables**

**Supplemental Table I. Participant demographics by eligibility for Part B (GCS use)**

| **Demographics** | **Not wearing GCS**  **n=321 (%)** | **Wearing GCS**  **n=77 (%)** |
| --- | --- | --- |
| **Median age (years)** | 62 (48-75) | 54 (36-75) |
| **Sex** |  |  |
| Female | 137 (43) | 32 (42) |
| Male | 184 (57) | 45(58) |
| **Ethnicity** |  |  |
| First Nations | 134 (42) | 22 (29) |
| Non-First Nations | 187 (58) | 55 (71) |
| **Division** |  |  |
| Surgical | 107(33) | 46 (60) |
| Medical | 203 (63) | 23 (30) |
| Obstetric | 11 (4) | 8 (10) |
| **Residency** |  |  |
| Urban | 121 (38) | 39 (51) |
| Rural | 198 (62) | 32 (42) |
| Interstate | 2 (1) | 6 (8) |

Abbreviation: GCS: graduated compression stockings. Data presented as n (%) or median (interquartile range); there were 49 prescriptions for GCS, however, 77 participants were wearing GCS when visual inspection was conducted.

**Supplemental Table II. Absolute contraindications and potential risk factors for Graduated Compression Stocking use**

| **Absolute contraindications** | **Participants prescribed GCS**  **n=49 (%)** | **Participants wearing GCS and included in Part B**  **n=46 (%)** |
| --- | --- | --- |
| Absence of foot or leg pulses | 1 (2) | 5 (11) |
| Leg Claudication | 0 (0) | 0 (0) |
| Lower limb pain on resting | 0 (0) | 0 (0) |
| Ankle brachial index <0.7 | 0 (0) | 0 (0) |
| Toe pressure <50mmHg | 1 (2) | 0 (0) |
| Significant arterial disease | 0 (0) | 0 (0) |
| Peripheral neuropathy | 1 (2) | 1 (2) |
| Lower Limb Pressure injury | 0 (0) | 1 (2) |
|  |  |  |
| **Risk factors for adverse events** |  |  |
| Prior lower limb amputation | 0 (0) | 1 (2) |
| Bunion present | 1 (2) | 5 (11) |
| Charcot Foot deformity | 0 (0) | 0 (0) |
| Other foot deformities hammer toes | 0 (0) | 0 (0) |
|  |  |  |

*Total of 3 participants in column 1 (n=49) prescribed with GCS, 1 participant had 2 contraindications, (neuropathy and low toe pressures)

**Supplemental Table III. Assessment of fit and sizing in participants wearing graduated compression stockings**

|  | **n=46 (%)** |
| --- | --- |
| **Legs Measured prior to fitting** | 26 (56) |
| **Changed size of GCS** | 2 (4)* |
| **Sizing assessment** |  |
| Fitted well (on initial visual inspection) | 28 (61) |
| Bunched behind knee | 7 (15) |
| Bunched at ankle | 0 (0) |
| Foot swelling due to bunching | 2 (4) |
| Toe section around ankle | 0 (0) |
| Toe section around foot | 1 (2) |
| Foot section folded up | 0 (0) |
| Leg section folded down | 2 (4) |
| GCS cutting into skin | 6 (13) |
| Excess stocking around knee | 0 (0) |
| Excess stocking around ankle | 4 (9) |
| Excess stocking around foot | 0 (0) |
| Excess stocking around toes | 2 (4) |
| GCS too big | 8 (17) |
| GCS too small | 3 (7) |
| Tight foot with loose leg | 2 (4) |
| Loose Foot with tight leg | 0 (0) |
| Foot too long | 2 (4) |
| Foot too short | 2 (4) |
| Foot to small | 1 (2) |
| Foot too big | 1 (2) |

*****Two participants stated they changed GCS size due to burning of the heel and cutting into the toes when wearing them.

**Supplemental Figure 1. Correlation between foot length or width and knee, calf or ankle circumferences**

A B

ρ=0.26

ρ=0.52

C D

ρ=0.27

ρ=0.47

E F

ρ=0.43

ρ=0.70
